# Stimulus-Specific Adaptation and Deviance Detection in the Rat Auditory Cortex

**DOI:** 10.1371/journal.pone.0023369

**Published:** 2011-08-10

**Authors:** Nevo Taaseh, Amit Yaron, Israel Nelken

**Affiliations:** 1 Department of Neurobiology, Silberman Institute of Life Sciences, Hebrew University, Jerusalem, Israel; 2 The Edmond and Lily Safra Center for Brain Sciences and Interdisciplinary Center for Neural Computation, Hebrew University, Jerusalem, Israel; Tokyo Medical and Dental University, Japan

## Abstract

Stimulus-specific adaptation (SSA) is the specific decrease in the response to a frequent (‘standard’) stimulus, which does not generalize, or generalizes only partially, to another, rare stimulus (‘deviant’). Stimulus-specific adaptation could result simply from the depression of the responses to the standard. Alternatively, there may be an increase in the responses to the deviant stimulus due to the violation of expectations set by the standard, indicating the presence of true deviance detection. We studied SSA in the auditory cortex of halothane-anesthetized rats, recording local field potentials and multi-unit activity. We tested the responses to pure tones of one frequency when embedded in sequences that differed from each other in the frequency and probability of the tones composing them. The responses to tones of the same frequency were larger when deviant than when standard, even with inter-stimulus time intervals of almost 2 seconds. Thus, SSA is present and strong in rat auditory cortex. SSA was present even when the frequency difference between deviants and standards was as small as 10%, substantially smaller than the typical width of cortical tuning curves, revealing hyper-resolution in frequency. Strong responses were evoked also by a rare tone presented by itself, and by rare tones presented as part of a sequence of many widely spaced frequencies. On the other hand, when presented within a sequence of narrowly spaced frequencies, the responses to a tone, even when rare, were smaller. A model of SSA that included only adaptation of the responses in narrow frequency channels predicted responses to the deviants that were substantially smaller than the observed ones. Thus, the response to a deviant is at least partially due to the change it represents relative to the regularity set by the standard tone, indicating the presence of true deviance detection in rat auditory cortex.

## Introduction

The auditory system processes a continuously changing auditory scene. The detection of violations to regularities in the sound stream may be critical for survival, and may play an important role in the formation of auditory objects (e.g. [1], [2]). Neural mechanisms of deviance detection have been extensively investigated, mainly using the oddball paradigm [3], [4]. In this paradigm, deviance is typically produced by presenting a rare (‘deviant’) stimulus against a background of a frequent (‘standard’) stimulus. In cat auditory cortex [5], [6], rat inferior colliculus [7], barn owl midbrain and forebrain [8], mouse and rat auditory thalamus [9], [10] and rat auditory cortex [11], [12], the responses of neurons to a tone are larger when that tone is deviant than when it is standard. This effect, called stimulus-specific adaptation (SSA), depends on the physical difference between the standard and the deviant stimuli, on the probability of appearance of the deviant tone, and on the inter-stimulus time interval [5], [6], [7]. SSA shares many properties with, (but is probably not identical to, [12], [13]) mismatch negativity (MMN), a component of the auditory event-related potentials (ERPs) that is elicited by deviant tones in humans [14].

Current research is somewhat ambiguous regarding the definition of SSA. The term itself emphasizes the adaptation of the responses to the standard tone. However, the hallmark of SSA is the large response to the deviant. This large response could be due to the fact that the deviant is rare and, therefore, the response it evokes is not adapted. But the deviant also represents a violation of the expectation to hear a standard. Indeed, the MMN literature emphasizes the responses to the deviant tone, and a substantial effort has been made to demonstrate that MMN is not (or not only) due to the rarity of the deviant, but is at least partially due to the violation of the regularity of the tone sequence caused by the presentation of the deviant [15], [16], [17].

Two types of sound sequence have been used to uncover the factors contributing to the responses to deviants. The first is the ‘deviant-alone’ control, in which the tone sequence consists of mainly silent trials, broken by the occasional deviant tone at the same probability as in the oddball paradigm (but otherwise in random positions) [15]. In these sequences, there is presumably no regularity to break, and therefore the response should reflect rarity alone, in contrast with the response to deviants with standards, that includes a potential contribution of deviance as well. However, the response to the deviant in the deviant-alone sequences may be large simply because the auditory system is stimulated overall at a much slower rate than in the oddball sequence. This problem is alleviated, but not fully solved, by the ‘deviant among many standards’ control [16], [17], which keeps the rate of deviant tone presentations low but replaces the standard tone presentations with a number of different stimuli, each with a low probability of appearance, thus canceling the special status of the deviant. Responses to the deviant in this case presumably reflect its rarity while controlling for the overall activation of the auditory system, and therefore serve as an appropriate baseline for establishing the presence of a component which is sensitive to deviance in the responses to deviants in the oddball sequences.

Studies of human ERPs using both types of control sequences strongly suggest that the MMN is indeed an index of true deviance detection. However, the assumptions underlying both types of control sequences are not transferable in a simple way from the study of gross potentials such as MMN to neuronal responses in auditory cortex. In this study, we examined deviance detection in auditory cortex of rats. To do so, we used a variety of stimulus conditions, including the control conditions used in human experiments. These data made it possible to disentangle the effects of tone rarity and the effects of tone deviance by using a simple model that fitted well the responses. The main result of the paper is the demonstration of a response component that is due specifically to deviance detection in rat auditory cortex.

## Results

We presented sound stimuli to the right ear of halothane anaesthetized rats and recorded local field potentials (LFP) and extracellular spiking activity from left auditory cortex using tungsten electrodes. Based on the responses to pure tones spanning the range of 1–64 kHz, two frequencies evoking large responses were selected for further study. The lower frequency was denoted f1, the higher was denoted f2, and they were selected such that the frequency difference between them, defined as Δf  =  (f2−f1)/f1, was 10%, 21%, 44% or 96%. These values correspond to 0.141, 0.275, 0.526 and 0.971 of an octave, respectively. We presented f1 and f2 in oddball sequences (as in [6]), but we also embedded them in several control sequences that have not been used in previous studies of SSA in animals. For the sake of clarity, we first discuss the responses to the oddball sequences at a single Δf, then introduce the control sequences, and finally discuss the effects of Δf and inter-stimulus interval on the responses. In order to reduce ambiguity, the word ‘frequency’ will refer in this paper only to the acoustic frequency of the pure tones. Other meanings of ‘frequency’ will be designated by ‘rate’ (of stimulus presentation) or ‘probability of occurrence’ (of a tone of a given frequency within a test sequence).

### Stimulus-specific adaptation

The oddball sequences consisted of 500 pure tone beeps (30 ms duration, 5 ms rise/fall time), presented at an inter-stimulus interval (ISI) of 300 ms unless explicitly stated otherwise. Two oddball sequences were used. In one sequence, 95% of the stimuli, at random, had frequency f1, and the other 5% had frequency f2, so that f1 was frequent (‘standard’) while f2 was rare (‘deviant’). This sequence is called ‘Deviant f2’. To determine whether the difference in the responses to the standard and to the deviant tones was due to their different probability of presentation or to the frequency difference between them, we used another sequence in which the roles of the two frequencies were switched, so that f1 was deviant and appeared in 5% of the trials, while f2 was the standard. This sequence is called ‘Deviant f1’. For comparison, we presented also a third sequence in which the two frequencies appeared with equal probability (half of the tone presentations each, randomly).

**Figure 1:**
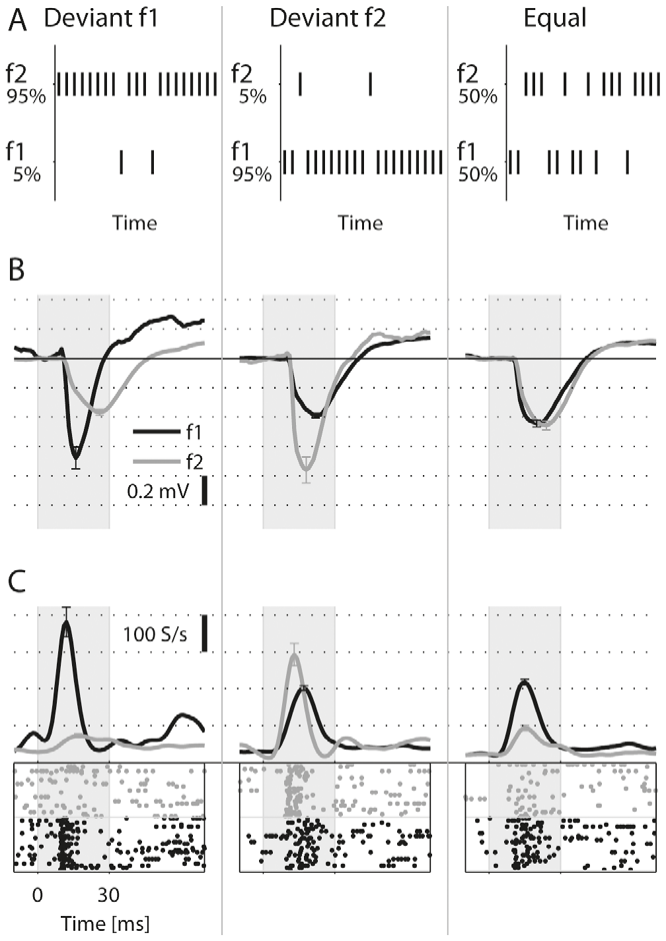
The oddball paradigm. A. A schematic spectrographic representation of the three basic sequences used in this study. In each trial, either f1 or f2 are presented pseudo-randomly according to their probability of occurrence. B. The average LFP responses in a typical recording site to the two frequencies of the paradigm in each of the sequences (f1 = 13.3 kHz, black, and f2 = 19.2 kHz, gray). The level was 30 dB attenuation (∼70dB SPL). Error bars: ± s.e.m., shaded interval: stimulus. C. MUA Responses at the same site. The raster plots show 25 presentations for each of the two frequencies, corresponding to 5% of the 500 tone presentations in the sequence. For the standard and equal-probability conditions, which had more than 25 presentations in a sequence, the 25 presentations were selected so that they represent the spike count distribution of all responses in the time window shown. The line graphs represent PSTHs smoothed by a 10 ms Hamming window.

Figure 1A describes schematically the two oddball sequences, as well as the equal-probability sequence. Figures 1B and 1C display the average LFP as well as the multiunit activity (MUA) recorded simultaneously from a typical recording site (f1 = 13.3 kHz, f2 = 19.2 kHz, corresponding to Δf of 44%, 70 dB SPL). In the ‘Deviant f1’ sequence, the LFP response to f1 (the deviant) was considerably larger than the response to f2 (the standard). On the other hand, in the ‘Deviant f2’ sequence, the response to f1 (now the standard) was smaller than the response to f2. The LFP responses to the two frequencies in the equal-probability sequence were comparable, and matched the corresponding responses to the same tones when standard, but were substantially smaller than the responses to the same tones when deviant. Thus, the LFP responses were similarly depressed by tones with probabilities of occurrence of 50% and 95%, but this depression did not generalize to other tones with probability of occurrence of 5%, at least for Δf = 44%.

In order to quantify the effect of presentation probability on tone response we used the contrast between the responses to the same frequency when it was standard and when it was deviant, called the ‘stimulus-specific adaptation index’ or SI [5]:
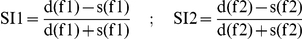



where d(f*i*) and s(f*i*) represent the peak responses to frequency f*i* when it was deviant and standard, respectively (see Methods for details of the measurement of peak responses for LFP and multiunit activity). For the LFP responses in Fig. 1B, SI1 was 0.27, and SI2 was 0.35. Both contrasts were positive, demonstrating the appreciable effect of stimulus probability on the sensory responses.

The MUA responses measured at the same site are displayed in Fig. 1C. Similarly to the LFP, the MUA response to each of the tones was smaller when standard than when deviant. Remarkably, while in the equal-probability sequence the MUA response evoked by f2 was substantially weaker than that evoked by f1, in the Deviant f2 sequence the MUA evoked by the deviant f2 was actually larger than that evoked by the standard f1, the opposite of what one would predict from the frequency selectivity of this site.

**Figure 2 pone-0023369-g002:**
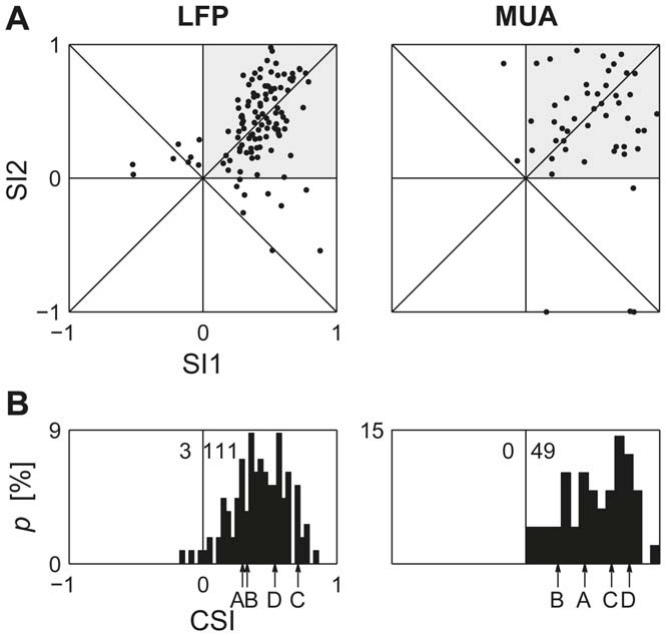
SSA indices for Δf = 44%. A. Scatter plots of SI2 vs. SI1 for all sets with Δf = 44%. B. Histograms of CSI. The numbers near the zero line indicate the number of CSIs smaller and greater than 0. The labels A – D mark the bins corresponding to the data in Figure 4.

Figure 2A shows the frequency-specific contrasts between the standard and the deviant responses, SI1 and SI2, for all the recording sites tested with Δf = 44%. As shown in Ulanovsky et al. [5], if the change in response size were due to a general decrease in the excitability of the neural signal (‘fatigue’), data would have SI1+SI2 = 0, corresponding to points along the reverse diagonal. In fact, in virtually all cases not only SI1+SI2>0 held, but also both SI1 and SI2 were individually positive. Thus, the example in Fig. 1C is typical.

The common contrast between the deviant and standard responses was used to characterize the average effect of adaptation for this specific pair of frequencies [6], [10]:
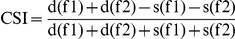



For the responses in Fig. 1, CSI = 0.31. The distribution of the common contrast CSI for all the data with Δf = 44% is shown in Fig. 2B. The CSI was essentially always positive, demonstrating the robustness of the SSA in rat auditory cortex.

### Controls for SSA

**Figure 3 pone-0023369-g003:**
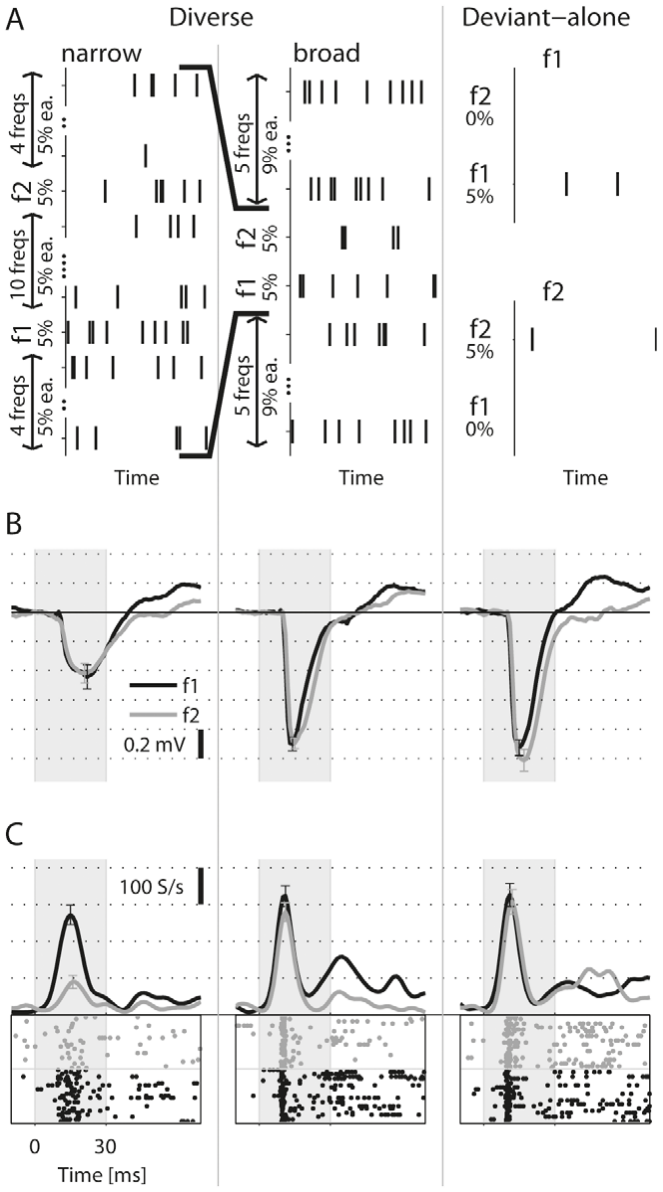
The control sequences. A. The three control conditions. In the ‘Diverse’ sequences, narrow and broad, the available frequencies were evenly spaced on a logarithmic frequency scale. The range of frequencies was narrow (about twice Δf) in the Diverse-narrow sequence, and wide (11Δf) in the Diverse-broad sequence. In the ‘Deviant-alone f1’ and ‘Deviant-alone f2’ sequences, the other 95% of the presentations were silent. B. The average LFP responses to frequencies f1 and f2 embedded in the control sequences (same recording site as Fig. 1). The responses in the two Deviant-alone sequences are superimposed. Error bars: ± s.e.m., shaded interval: stimulus. C. MUA Responses at the same site.

The main goal of this study was to elucidate the effects of the standard tone on the deviant responses. We therefore used several control sequences in which the two tested tones had the same probability of occurrence as the deviant in the oddball sequences, while the other tone presentations in the sequence, the ‘context’, had different frequency compositions. Figure 3A describes the control sequences.

The first control consisted of tones of 20 different frequencies, including f1 and f2. Each frequency was presented with an equal probability of 5% in a pseudo-random order, so f1 and f2 were as rare as the deviants in the oddball sequences, but so were all the other frequencies. The 20 frequencies were equally distributed on a logarithmic scale that spanned about twice Δf, with f1 and f2 symmetrically positioned (the 5^th^ and 16^th^ frequencies among the 20). Thus, the frequencies were densely packed in a relatively narrow frequency band. For Δf = 44% the frequency ratio between adjacent frequencies was 3.37%. This sequence will be referred to here as the narrowband diverse sequence, or ‘Diverse-narrow’ in short.

The second control was similar to the diverse-narrow sequence, but the frequency interval between adjacent tones was Δf (the frequency separation between the test frequencies f1 and f2). With intervals of Δf = 44%, a series of 20 frequencies would span far more than the audible frequency range of the rat. Therefore, we used tones of only 12 different frequencies for this control. Frequencies f1 and f2 were each presented with a probability of 5% to match that of a deviant in the oddball sequences, but the other 10 frequencies were presented with a probability of 9% each. This distribution resulted in an asymmetry between f1 and f2 on the one hand and the other frequencies in the sequence on the other hand, but the number of different frequencies was large enough to mask this asymmetry [16]: in humans, it is sufficient to ‘distribute’ the probability of the standard among only 4 different stimuli in order to remove the contribution of deviance to the responses, and we observed similar results in rats in preliminary experiments (data not shown). This sequence will be called the broadband diverse sequence or ‘Diverse-broad’ in short.

In terms of tone probability and lack of regularity the Diverse-narrow and Diverse-broad controls are comparable. However, the responses to the oddball sequences were expected to depend on frequency difference between the standard and the deviant. The two controls made it possible to study the effects of frequency separation on SSA.

The third control condition was based on the ‘Deviant-alone’ control of the MMN literature [15], and there were two such sequences, one for each of the two main frequencies. In the ‘Deviant-alone f1’ sequence, tones of frequency f1 were randomly presented in 5% of the presentation intervals, as in the Deviant-f1 oddball sequence, but the standard presentations were replaced by silence. Similarly, the ‘Deviant-alone f2’ sequence presented the deviant f2 against a silent background. These two sequences matched the oddball sequences in terms of rarity and uniqueness of the rare stimulus (unlike the diverse sequences), but unlike all other sequences, in the Deviant-alone sequences there was no interaction with any other stimulus.

Figure 3B shows the LFP responses to f1 and f2 in the three control sequences, recorded from the same recording site as the data presented in Figs. 1B and 1C. The responses to f1 and f2 in each of these sequences were similar. The responses in the Diverse-narrow sequence were smaller than those of the Diverse-broad or the Deviant-alone sequences, demonstrating the presence of cross-frequency adaptation. On the other hand, the responses in the Diverse-broad and the Deviant-alone sequences were comparable. The MUA responses shown in Fig. 3C showed similar trends, except that cross-frequency adaptation in the Diverse-narrow sequence was stronger for frequency f2 than for frequency f1, possibly because f1 was closer to the best frequency of the MUA at this site.

**Figure 4 pone-0023369-g004:**
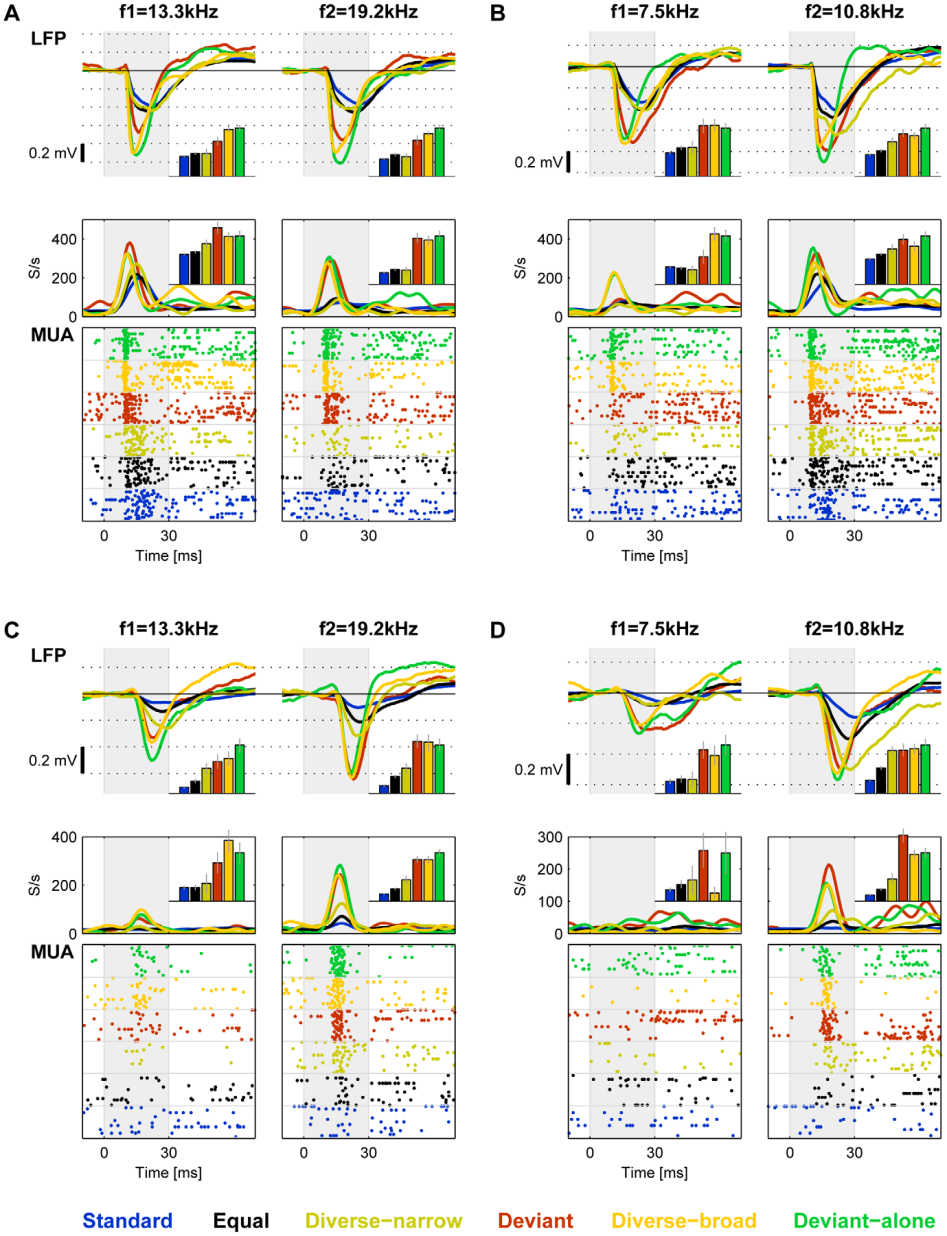
Typical responses to all conditions (Δf = 44%). A. Responses of LFP and MUA recorded at the same site as Figs. 1 and 3 to f1 and f2 in all stimulus conditions, sorted by frequency. Insets: the amplitudes of the responses, normalized by the responses at the corresponding deviant-alone condition. The raster plots display only 25 representative responses in the standard and equal-probability conditions, as in Fig. 1. B. Responses recorded in the same recording site as A to sequences with different f1 and f2. C,D. Additional examples of the responses to all sequences, recorded from another rat.

We therefore had seven different sequences (Deviant-f1 and -f2, Equal probability, Diverse-narrow and -broad, and Deviant-alone f1 and f2) that tested both f1 and f2 in six conditions. The collection of responses to one frequency in all six conditions is termed ‘a set’ below. Figure 4A re-plots the responses from Figs. 1 and 3, grouped by frequency rather than by presentation sequence. It therefore illustrates the influence of context on the responses to tones of each of the two frequencies. Figure 4B shows responses recorded from the same recording site as in Fig. 4A, but to another pair of frequencies. Two other typical cases from another animal are shown in Fig. 4C and 4D, all with Δf = 44%.

Note that the peak response of the MUA to f1 in the Deviant condition in Fig. 4B occurred about 40 ms after stimulus onset, while the peak responses to the other conditions occurred about 20 ms after stimulus onset. This was an effect of the rather long window used here to identify the peak responses (see Methods for justification). Because the same procedure was used in all conditions, such long windows could increase the variability in the data, rendering the conclusions more conservative.

**Figure 5 pone-0023369-g005:**
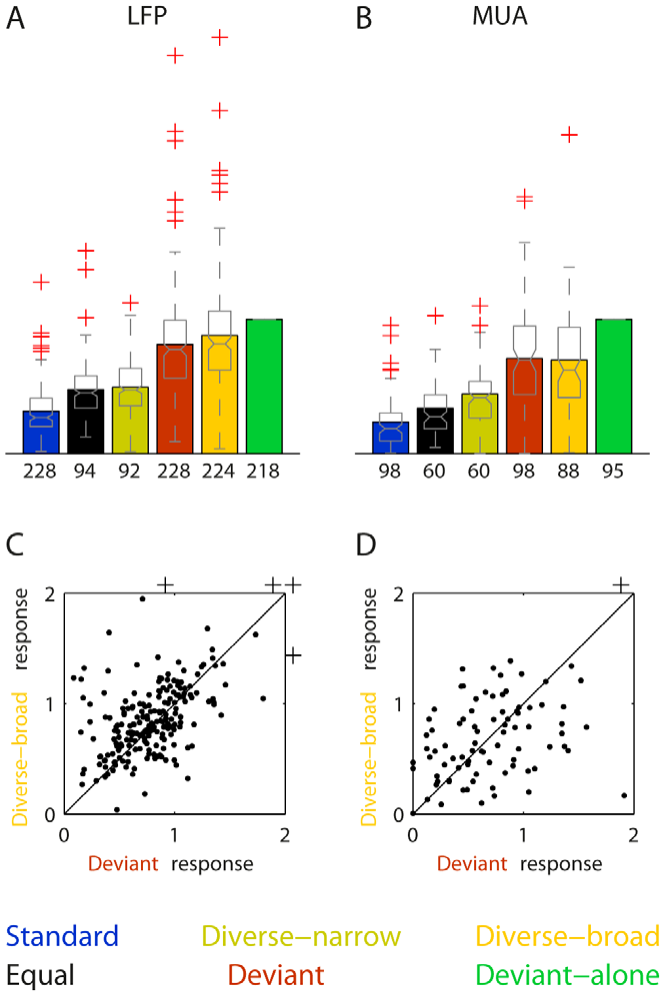
Summary of the responses to all conditions (Δf = 44%). A and B. Average (bar plots) and distributions (summarized as box plots) of the responses (normalized with respect to the corresponding deviant-alone response) to both frequencies in each of the six conditions, in all sets recorded with Δf = 44%. The number of cases included in each column is displayed underneath it (not all sets included all the six conditions). A. LFP, including 114 sets recorded from 60 individual sites in 22 rats. B. MUA, including 49 sets recorded from 35 sites in 18 rats. C and D. The normalized response to in the Deviant condition (abscissa) versus the normalized response to the same tone in the Diverse-broad condition (ordinate). Each point represents one of the main frequencies (either f1 or f2) of a set in a specific recording site. C. LFP, (r = 0.62, df = 212, p<<0.001). D. MUA (r = 0.40, df = 83, p<0.001).

The pattern of response was quite similar between sets across animals and recording sites. Figure 5 shows a summary of the responses to all six conditions for all the data with Δf = 44%. In order to account for general differences in response amplitude across animals and recording sites, the responses in each recording site and each frequency were normalized to the response to that frequency in the deviant-alone condition of the same set, since this is presumably the least adapted response. When a tone was rare and well separated spectrally from other stimuli in the sequence, as in the deviant and diverse-broad conditions, the responses it evoked remained relatively high. On the other hand, when the tone was presented often (as in the Standard and Equal-probability conditions), or mixed with many tones with nearby frequencies (as in the Diverse-narrow condition), the responses it evoked were smaller. A 1-way ANOVA on all six conditions showed significant effect of condition on responses. However, post-hoc comparisons failed to show a significant difference between the three conditions with large responses (Deviant, Diverse-broad and Deviant-alone).

The approximate equality of the responses in the Deviant and the Diverse-broad conditions is particularly important for the interpretation of these findings in the Discussion. Figures 5C and 5D display the LFP and MUA responses in the Diverse-broad condition against the responses in the Deviant condition in each recording site separately. Both responses are normalized to the Deviant-alone condition. The responses in the Deviant condition were correlated with the Diverse-broad in the same recording locations for both LFP (r = 0.62, p = 7*10^−24^) and for MUA (r = 0.4, p = 1.5*10^−4^). Interestingly, in some recording locations the Deviant responses were larger than the responses in the Deviant-alone condition (normalized Deviant responses greater than 1, LFP: 50/214, 23%; MUA: 18/85, 21%). In many recording locations, the Deviant responses were larger than in the Diverse-broad condition (points below the diagonal of Figs. 5C and 5D, LFP: 83/214, 39%; MUA: 43/85, 51%). In some recording locations, both conditions held (LFP: 29/214, 14%; MUA: 17/85, 20%; see for example the MUA responses in Fig 4A, 13.3 kHz and Fig. 4D, 10.8 kHz).

### Dependence of SSA on Δf

**Figure 6 pone-0023369-g006:**
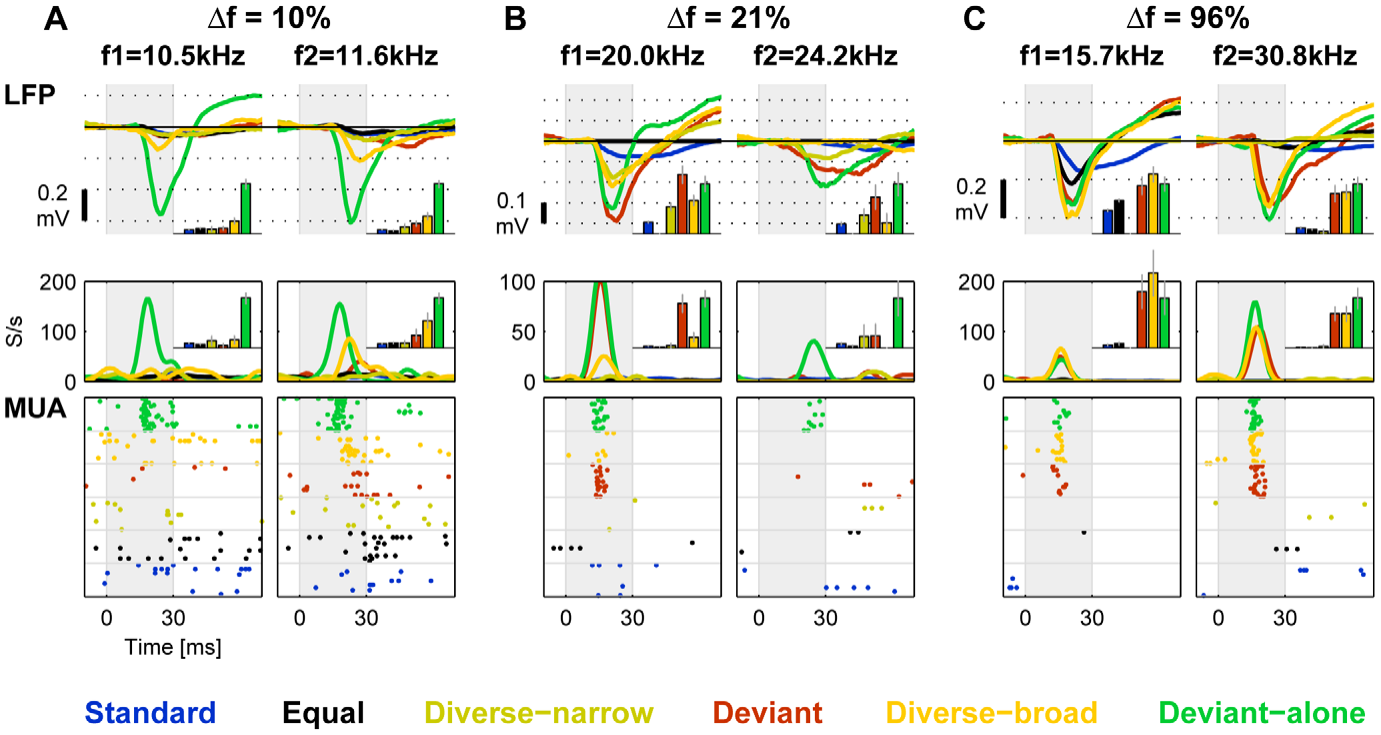
Responses as a function of Δf. A. LFP and the corresponding MUA responses to the two frequencies in a paradigm with Δf = 10%. B. Δf = 21% (from another animal). C. Δf = 96% (another animal). Same conventions as in Fig. 4.

To assess the bandwidth over which cross-frequency adaptation contributes to SSA, we presented the same types of sequences with Δf''s of 10%, 21% and 96%. Figure 6 shows typical responses to stimulus pairs with these three values of Δf, recorded from three different animals. With Δf = 10%, all conditions except Deviant-alone showed strong adaptation, probably due to proximity between different frequencies in the same sequence. The least adapted responses occurred in the Diverse-broad condition that spanned the broadest frequency band. Nevertheless, the LFP response to each frequency when deviant was stronger than the response to the same frequency when standard; the same occurred for the MUA responses to f2 (11.6 kHz). With Δf = 21%, the contrast between the responses to the same frequency in different conditions was larger and the pattern of responses became qualitatively similar to that of Δf = 44%. With Δf = 96% the two frequencies were presumably too far apart to elicit cross-frequency adaptation, except maybe for the Diverse-narrow condition where frequencies were still rather densely packed.

**Figure 7:**
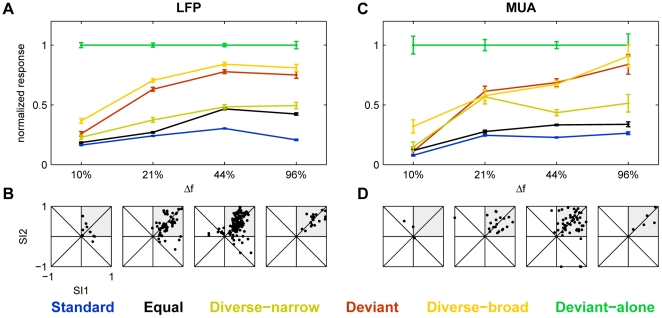
Summary of the responses at all Δf. A. The population average of normalized LFP responses to the six conditions for four different values of Δf. B. Scatter plots of SI2 vs. SI1 for LFP data from all recording sites as a function of Δf. C,D. Same as A,B, but for MUA data. The scatter plots of SI2 vs. SI1 at Δf = 44% are those appearing in Fig. 2A, and have been replotted here as well for completeness.

These data, for all values of Δf, are summarized in Fig. 7. The responses were normalized to the Deviant-alone responses of the same set (as in Fig. 5). Clearly, cross-frequency adaptation strongly affected the responses for Δf = 10%, and didn't affect much the responses at Δf = 44%. At Δf = 21%, there was some decline in the size of the responses, suggesting that this was the effective bandwidth of cross-frequency adaptation. SSA was nevertheless evident in both LFP and MUA at all Δf, even in the strongly reduced responses at Δf = 10%.

### Dependence of SSA on ISI

**Figure 8:**
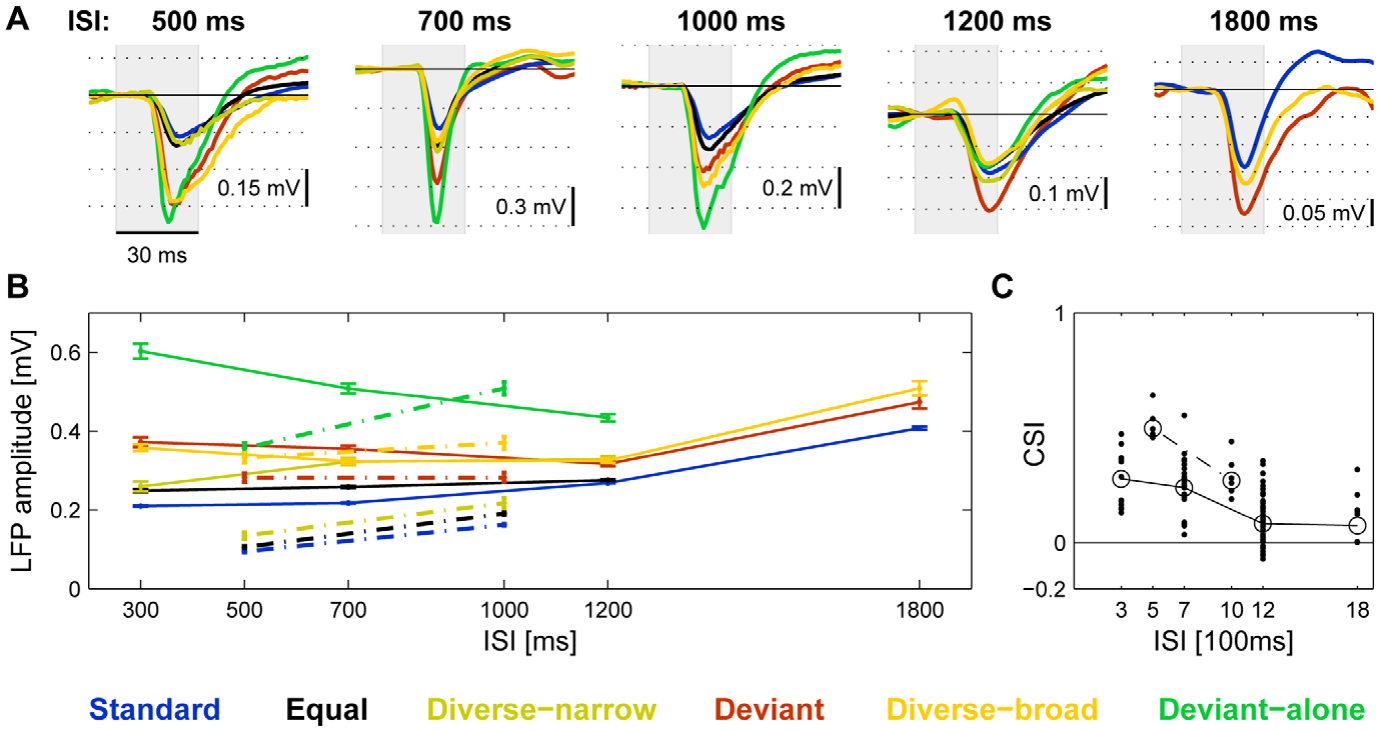
Respones as a function of ISI. A. Average LFP responses at five different ISIs. The examples, from different animals, show the responses to the low frequency (f1) in each pair. B. The population average of the responses to different ISIs for the two groups of rats (continuous and dashed lines) used in testing the effects of ISI. The error bars are s.e.m. For more details, see text. C. The distributions of the individual CSIs at each ISI. Circles are ‘common’ averages, computed as the sum of all numerators of contrasts in each ISI value, divided by sum of all denominators.

The dependence of SSA on ISI was studied with Δf = 44%. In two rats, responses were collected for ISI of 500 ms and 1000 ms. In another group of four rats ISIs of 300, 700, 1200 and 1800 ms were used. SSA was evident at all ISIs, as illustrated by the responses in individual examples (Fig. 8A): in all of these cases, the responses to a frequency when Deviant (red) was larger than the responses to the same frequency when Standard (blue). The population average for each group (Fig. 8B) showed a decrease in SSA as ISI increased (Fig. 8C), but SSA was significant even with ISIs of 1800 ms both in individual recording sites and in the population average. To check the significance of the different apparent trends of response magnitudes in the two groups of rats, a 3-way ANOVA was performed, the factors being the groups of rats (two levels), conditions (six levels), and short vs. long ISIs (two levels: in the first group 500 ms was considered as short ISI, 1000 ms as long ISI, while in the other group we coded 300 ms and 700 ms as short ISIs, 1200 and 1800 ms as long ISIs). The main effect of ISI on the size of LFP responses was not significant (F(1,868) = 3.31, p = 0.07), and even the interaction between ISI and the group of rats was only borderline significant (F(1,866) = 4.35, p = 0.04). We conclude that the difference in trends in the two groups, even if present, is small.

### Modeling SSA

The data suggest that the responses to tone sequences are shaped by ‘adaptation channels’ whose width is about 20% of their center frequency. To check whether this mechanism is sufficient to account for the data, we turned to modeling. The model describes the effects of tone presentations at all frequencies on the responses to tones at the center frequency, 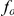, of the adaptation channel.

The model is inspired by models of adaptation by depletion of synaptic resources [18]. The relationship between the model which is heuristically developed below and a formal model of resource depletion is clarified in Text S1. The model assumes that each tone presentation depletes synaptic resources available to produce responses. The interplay between synaptic depletion and recovery during the silence between two stimuli (considered as fixed here, since only the data with ISI = 300 ms was modeled) results in a steady state response after a few stimulus presentations [19]. The steady state response to tones of frequency *f_0_* within a sequence composed only of tones of frequency *f_0_*, normalized by the unadapted response, will be denoted by *B<1*. The responses to very rare tones at frequency *f_0_* embedded in a sequence of tones of a frequency *f* that is different from *f_0_* should still be adapted, but only partially. To capture this frequency dependence, the model posits that the normalized response to a tone at frequency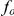 after a long sequence of tones of frequency *f* is 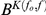, where the kernel *K* is a (non-normalized) Gaussian,



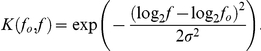



The half-width of the cross-frequency adaptation band, 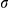, is the main parameter of interest of the model. For *f = f_0_*, the *K(f_0_,f_0_)* is 1 and we recover the steady state response level *B*. As *f* gets farther away from *f_0_*, *K(f_0_,f)* decreases to 0 so that the normalized response level 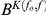 approaches 1 (the unadapted response).

The sequences we used consisted of tones of multiple frequencies. To combine the effects of all of these frequencies multiplicatively, the average contribution of all the presentations of tones at frequency *f* that appeared with probability *p_f_* in the sequence was modeled as 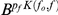. The overall adaptation depth is a product of the ideal unadapted response, 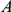, and the adaptation contributed by each of the frequencies that appeared in the sequence:



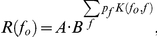



where the sum is taken over the frequencies of all tones in the sequence. The last formula should be considered as an estimate of the steady-state adapted response to frequency 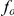 during a ‘typical’ stationary sequence of stimuli, in which the different frequencies were randomly intermixed with the overall proportions given by 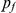. The model has three parameters: 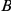, 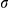 and 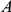.

To motivate the comparison that will be made between the model and the data, we start by giving a semi-quantitative description of the behavior of the model applied to the type of sequences used here. Obviously, the responses that are predicted by the model are inversely related to the sum in the exponent, which will be called ‘the adaptation load’. In the Standard, Equal-probability and Diverse-narrow conditions, it is clear why the adaptation load is relatively large: *f_0_* and/or nearby frequencies, for which the kernel is large, are highly probable. The interesting cases consist of the three conditions that give rise to large responses: Deviant-alone, Diverse-broad, and Deviant (in a decreasing order of the average responses).

**Figure 9:**
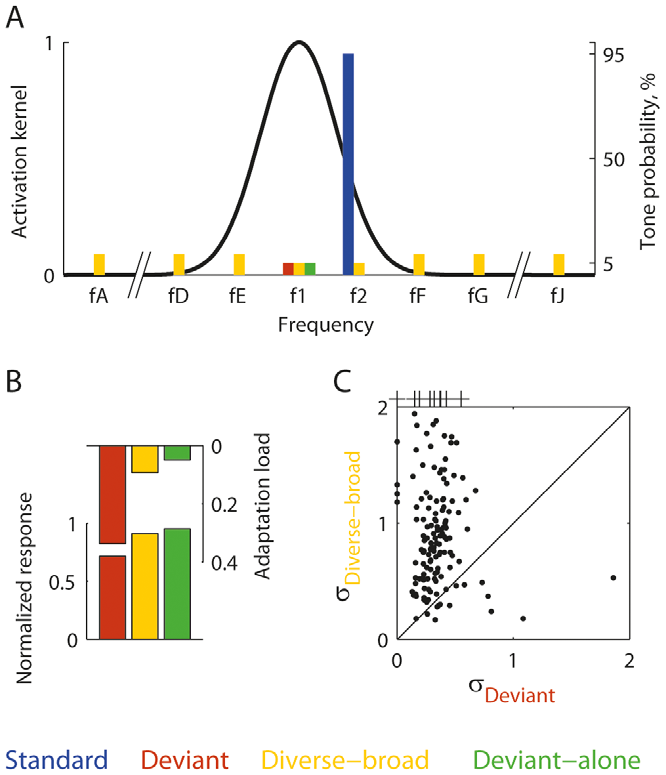
A simple adaptation model. A. A schematic plot of the adaptation channel centered on *f_o_* = f1 with σ = 0.34 (left ordinate) and three of the sequences that included this frequency with probability of 5% (right ordinate), all three with Δf = 44%. The Deviant f1 sequence (probability of f1 marked in red) included also tones at frequency f2 with a probability of 95% (blue). Frequency f2 lies within the effective range of the kernel centered on f1, thus causing a significant amount of adaptation. The Diverse-broad sequence includes f2 with probability of 5% and 10 other frequencies with a probability of 9% each (yellow). These additional frequencies are marked here fA to fJ (some of them are omitted from the figure due to space limitations). The Deviant-alone f1 sequence (green) does not contain any other frequency. The bars representing tone probabilities at f1 and f2 for the different sequences are shown side by side. B. Upper bars - the adaptation load for the three conditions, 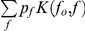. Lower bars – the predicted normalized steady-state response. Note that the Deviant response is appreciably smaller than the Diverse-broad response, which is about the same as the Deviant-alone response. In the measured data, the Deviant and Diverse-broad responses had about the same size. C. The half-width of the kernel computed for each of the LFP recording sites with Δf = 44% when derived from Deviant responses (abscissa) plotted against the half-width of the kernel derived from Diverse-Broad responses (ordinate). The normalized adaptation load when the center frequency was the Standard was assumed to be 0.90, the minimum possible. The results were virtually the same when the normalized adaptation load for the standard was assumed to be maximal, 0.95.

The adaptation load is least (and therefore the predicted responses largest) in the Deviant-alone condition, where it is equal to 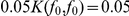, 0.05 being the probability of the deviant, and 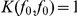 (see Figs. 9A, green bar and 9B; in Fig. 9A the adaptation channel is centered on frequency f1). In the Diverse-broad and Deviant conditions the adaptation load includes this term as well, but has additional contributions.

In the Diverse-broad condition, the additional terms in the adaptation load span a relatively large frequency band. If the width of the kernel *K(f_0_,f)* is on the order of the spacing between the frequencies used in the sequence, only a few terms will contribute significantly to the adaptation load, because of the shape of the kernel. For example, if the width of the kernel is *σ* = 0.34 (which is a typical value for the data, see below) and the spacing between frequencies is Δf = 44%, only two additional terms will contribute significantly to the adaptation load, one frequency on each side of the center frequency (Fig. 9A, yellow bars: the two contributing frequencies are f2, with probability 0.05, and fE, with probability 0.09). The adaptation load in the Diverse-broad condition is therefore approximately 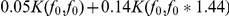. Thus, the normalized response in the Diverse-broad condition is expected to be about 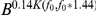. Since in the majority of cases the normalized response in the Diverse-broad condition is smaller than 1 (Figs. 5C, 5D), it follows that 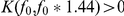



must hold – in other words, the width of the adaptation channel is non-zero.

In the Deviant condition, the additional term in the adaptation load is solely due to the standard, and the adaptation load is 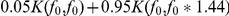 (Fig. 9A, red and blue bars; Fig. 9B). The normalized response to the Deviant is therefore 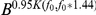. Intuitively, the adaptation load in the Deviant case will be larger than that in the Diverse-broad condition, because in the Deviant condition, all sound presentations that contribute to the adaptation have been brought closer to the deviant frequency (the blue bar is as high as the sum of probabilities of all the yellow bars except for the one at f1). In fact, under the assumptions we made about the rate of decrease of K, the model makes the prediction that the response in the Deviant condition should be smaller than in the Diverse-broad condition, and by appreciable amounts: with the approximations made above, if the normalized response in the Diverse broad condition is 0.9, the normalized response in the Deviant condition is expected to be less than 0.6 (Fig. 9B).

The normalized responses in the Deviant and in the Diverse-broad conditions can be used to derive estimates for the width of the adaptation channel. For that purpose, it is necessary to estimate B, which we do by using the normalized response to the Standard condition. In the Standard condition, *f_0_* is presented in a probability of 0.95, complemented by the other frequency, Δf apart. The adaptation load is therefore 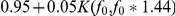, and the normalized response is therefore 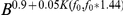. The kernel is smaller than 1, so the normalized response to the Standard lies between *B* ^0.95^ and *B* ^0.90^. The calculations below set the Standard responses to *B* ^0.90^; setting the Standard responses to *B* ^0.95^ resulted in practically the same values. We estimated the width of the adaptation channel using the responses to the Diverse-broad condition and separately using the responses to the Deviant condition. In both conditions, we calculated the predicted normalized response as a function of the width of the adaptation channel, and selected the width that corresponded exactly to the measured response. For this analysis, we used only the data for which the responses in the Deviant condition were smaller than the responses in the Diverse-broad condition, and both were smaller than in the Deviant-alone condition (these conditions are necessary for a solution to exist with *B*<1 and 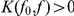). It should be remembered, however, that these cases comprise only about half of the total data (see Fig. 5).

Figure 9C shows the two estimates of the width of the adaptation channel plotted against each other for the LFP data collected with Δf = 44%. Clearly, the Diverse-broad responses required a consistently wider adaptation channel than the Deviant responses. To recapitulate, this discrepancy is due to the experimental findings that the responses in the Diverse-broad condition are smaller than in the Deviant-alone condition (requiring a large enough adaptation bandwidth), but are about the same size as in the Deviant condition (an equality that can hold only for small adaptation bandwidth). Thus, these two findings are incompatible with a model of adaptation in narrow frequency channels.

The analysis shown in Fig. 9 used only a small part of the data – only data with Δf = 44%, using the responses to only four out of the six conditions in which the tones were tested, and only half of the points. Furthermore, the parameters of the model were estimated suboptimally. In the rest of this section, we overcome these limitations. We first fitted a model to all the data of each recording site. As will be seen below, these models gave rather good fits to the data, but their error, although close to, was often larger than the best possible error. We reasoned that this bias had to do with the two conflicting requirements discussed above: the reduction in the responses to the Diverse-broad condition relative to the responses in the Deviant-alone condition, but the almost equality of the Diverse-broad and Deviant responses. We therefore removed each of the test conditions in its turn from the training set and refitted the models, using them to predict the responses in the left-out condition. As expected, removing either the Diverse-broad or the Deviant conditions resulted in the greatest changes in both the quality of the fit and in the estimated width of the adaptation channel, and also with the largest deviations between predictions and measured responses to the left-out conditions.

We fitted the model separately in each individual recording site, using all the data recorded in that site, including all frequency pairs at all Δfs. The discrepancy between the model and the actual responses was measured by the squared differences between each of the measured responses and the model prediction of that response. These discrepancies were added over all the responses to each of the two frequencies in each of the six conditions (which differed in the probabilities of the tone, its accompanying tones in the sequence, and the frequency separation between these tones) in all the sets that were presented at that recording site (up to 48 different responses, since not all recording locations were tested with all conditions and Δfs). To account for the different number of stimulus presentations that were used to determine each response, each term was weighted:



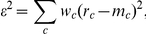



where the sum is over up to 48 terms, *r_c_* is the measured response in each condition, *m_c_* is the model prediction for that condition, and *w_c_* is the weight (square root of number of stimulus presentations at that condition).

The resulting total weighted squared error 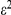 between the model response and actual response was then minimized in the following way. For each value of 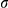, the half width of the adaptation channel, 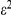 was minimized to produce the optimal 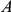 and 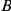 (using the fit function from the Matlab curve fitting toolbox). This made 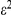 into a function of 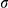 only. A one-dimensional search was used to find the value of 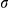 that minimized 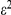. We didn't allow 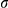
to decrease below 0.05 or increase above 4.0. Cases in which 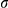
reached one of these boundaries were omitted from further analysis (7/91 LFP sites, 8%; and 6/43 MUA sites, 16%). All further analysis considers only recording sites in which the parameters were successfully estimated.

The parameters 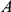 and 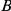 were also determined as part of the fitting process. The parameter 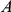 is a scale parameter which depends on the overall size of the responses. Since we applied the model to responses that were normalized, each to its corresponding deviant-alone responses in each set and frequency, 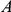 was expected to be, and indeed was, around 1, independently of recording site and frequency. *B* was essentially equal to the normalized response to the standard, since the exponent in the defining formula of the model in that case is very close to 1. Neither parameter is further analyzed here.

**Figure 10:**
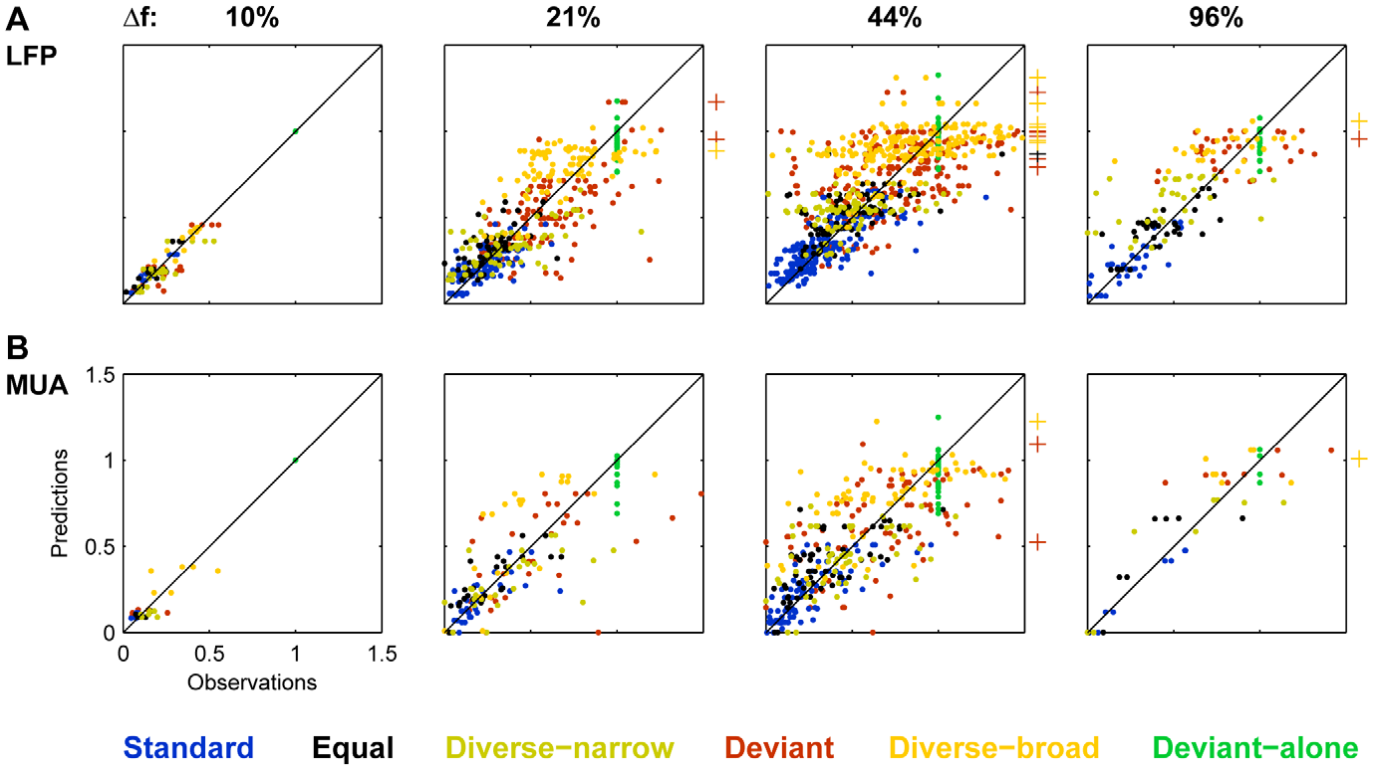
A comparison between the model predictions and the experimental observations. Scatter plots of all the measured responses (abscissa) versus the corresponding fits from the model based on all the data (ordinate) for each value of Δf. A. LFP data. B. MUA data.

The model accounted reasonably well for the data, given its simplicity. Figure 10 plots the fitted responses against the measured responses for all the recording sites and all types of sequences. Clearly, the model qualitatively captured the main aspects of the data: the fitted values to the Deviant-alone, Diverse-broad and Deviant (in green, yellow and red, respectively) were larger than to the other conditions. Also, overall, the individual fitted values scattered around the diagonal, as they should.

In order to judge the goodness of fit of the model more quantitatively, we compared the mean error of the model,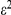, with the weighted sum of squared standard errors of the mean responses



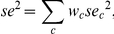



where the sum is over the same conditions as 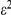, *w_c_* denotes the same weights used in calculating 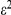, and *se_c_* is the standard error of the measured response, *r_c_*. This expression is an estimate of the error of the best possible model for the responses, in which the response to each condition is fitted by its observed mean value.

**Figure 11:**
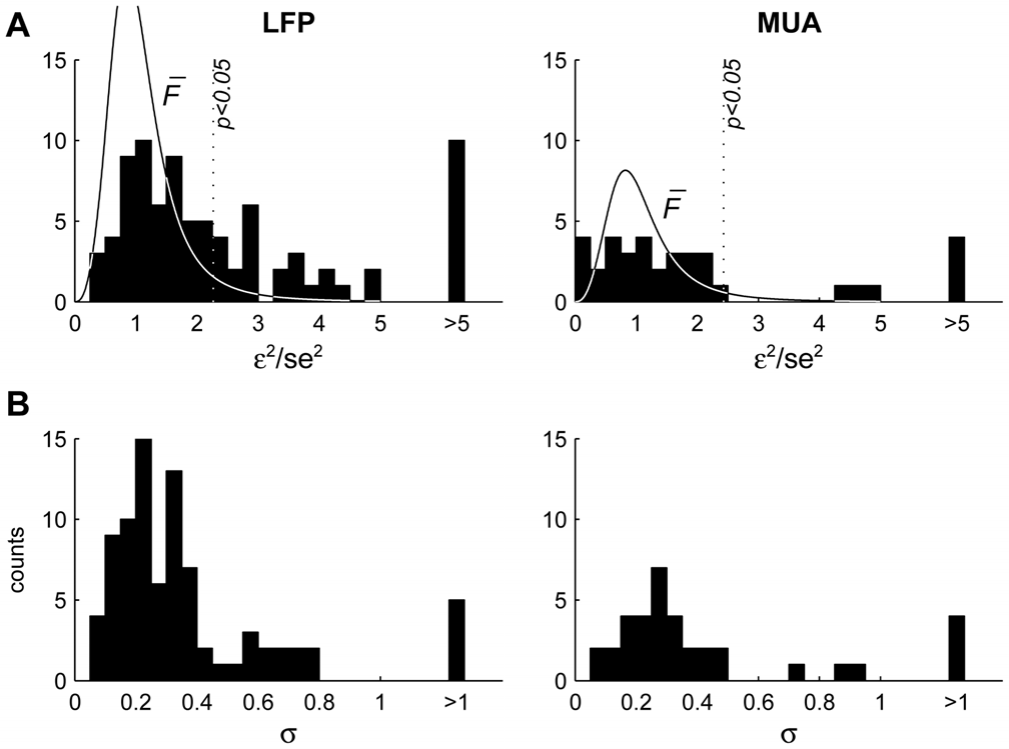
Model error and half-width of the adaptation channel. A. Distribution of the sum of squared errors between responses and fitted values, divided by the sum of squared s.e.m of the responses. The terms of both sums were weighted by the square root of the number of presentations of the corresponding stimulus. For comparison, the thin line indicates the average of the F(n,n) distributions associated with each of the sites, where n, the number of degrees of freedom, is the number of responses that were fitted by the model at that site. Vertical dotted line - the 0.05 critical value of the average F distribution. The majority of the models (51/84 of the models fitted to LFP responses, 61%, and 28/69 of those fitted to MUA responses, 81%) had an error ratio below this limit. B. Distribution of the half width of the adaptation channel, 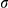, in all recording sites at which the fitting procedure converged successfully.

Figure 11A shows the histograms of the ratios between the fit error,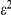, and the sum of squared standard errors of the responses, for all the relevant recording sites. The curves plotted on top of the histograms show the average expected distribution of the same ratios under the assumption that the model error is the minimal possible: since these are ratios of variances, assuming approximate Gaussian distribution for the errors, they should roughly follow an F(n,n) distribution with n, the number of degrees of freedom being the number of responses that were fitted by the model in each site. The average of all the F distributions for the modeled sites, and the corresponding 95% critical value, should give a rule of thumb for the goodness of fit. For the LFP data, 61% of the cases had error ratio below the critical value of the average F distribution, and for the MUA data, 81% of the cases had error ratio below the critical value. Thus, this 3-parameter model fitted much of the data quite well, although it also showed a consistent bias.

We were mostly interested in the estimated half-widths of the adaptation band, 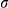. The distribution of 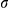 for the different sites is shown in Fig. 11B. For the LFP responses, the median of 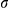 was 0.27 octave, with interquartile range of 0.19 – 0.40 octave. For the MUA responses, the median was 0.29 octave, with an interquartile range of 0.21 – 0.43 octave. Thus, the model suggests that in as much as SSA is due to adaptation in narrow frequency channels, significant across-frequency adaptation should occur once the tones are within 1/3 octave of each other.

Having established that the model produced a reasonable approximation to the data, we analyzed the fit in more details using the leave-one-out procedure described above. The general trends were similar for LFP and MUA data; the description below summarizes the main findings of the leave-one-out procedure, while leaving the interpretation to the Discussion.

**Figure 12:**
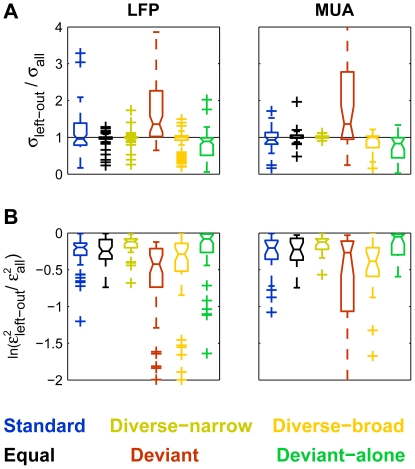
Comparing model error and half-bandwidth between all-data and leave-one-out models. A. Distribution of the ratios of the widths of the adaptation channel derived from a model for which responses to one condition were left out of the training data, and the width derived from a model that was fitted to all the data. The colors indicate the left-out condition. B. Distribution of ratios between the sum of squared errors of the model with the corresponding condition left out and the sum of squared errors of the model that was fitted to all the data (sums of squared error computed for the training data only).

The fits in the leave-one-out conditions had bandwidths and errors that differed from those of the fit to the whole data. Figure 12A shows the distribution of ratios of the adaptation bandwidths in the leave-one-out models and in the models fitted to all the data. Removing the Deviant responses from the training data resulted in a consistent increase in the fitted bandwidth. An opposite effect was evident when removing the Deviant-alone or the Diverse-broad responses from the training data, resulting in an overall decrease of the bandwidth of the adaptation channel. Such consistent effects were not seen for any of the other conditions.

The mean error over the training data was lower, as expected, when part of the data was left out (Fig. 12B). The two conditions that reduced the error most when left out, relative to the fit using the full data, were the Deviant and Diverse-broad conditions. In fact, the reduction in error when removing the Deviant responses from the fit was if anything larger than when removing the Diverse-broad responses (one-tailed t-test, p<0.05 for LFP, ns for MUA).

The predictions of the leave-one-out procedures showed some consistent bias relative to the actual measurements. A 2-way ANOVA on condition and Δf confirmed that the bias showed a significant effect of condition (LFP: F(5,1735) = 31, p∼0; MUA: F(5,615) = 12,p∼0), a non-significant main effect of Δf (LFP: F(3,1735) = 0.99, n.s.; MUA: F(3,615) = 0.95, n.s.), but a highly significant interaction between condition and Δf (LFP: F(15,1735) = 4.9, p∼0; MUA: F(15,615) = 2.38, p = 0.0024).

**Figure 13:**
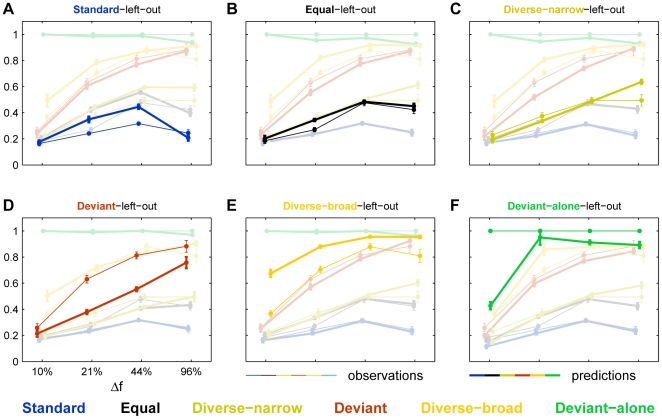
Comparing the leave-one-out model predictions with experimental observations. A-F. Comparison of the average LFP responses at the six stimulation conditions and four values of Δf (thin lines, the same in all panels) with the predicted responses from models in which one condition (denoted at the title of each panel) was left out of the training data (thick lines). Error bars: s.e.m. The data of the left-out condition is displayed in saturated color, while the data for the conditions that were part of the training set are displayed in faded colors.

Figure 13 compares the average measured responses with the predicted responses based on each of the leave-one-out models for the LFP responses. The general pattern of results is very similar for the MUA models. In each panel, the full-colored lines compare the measured (thin line) and predicted (thick line) responses to one left-out condition (indicated both in the title of the panel and through the color code). The pastel-colored lines show the same information for the five conditions that have been used to estimate the parameter of the model. As a rule, these responses are well-estimated, as expected from the ability of the model to account well for the data.

Taking out the responses in the Equal condition (Fig. 13B) and the diverse narrow condition (Fig. 13C) did not affect much the predictions, which fitted the measured responses very well on average. Taking out the Standard responses from the training set (Fig. 13A) resulted in over-prediction of these responses (thick blue line, representing the predictions, is overall above the thin blue line, representing the average responses). Conversely, taking out the Deviant alone responses from the training set resulted in predictions that were on average too small (Fig. 13F).

Most importantly, there were consistent consequences to removing the Deviant or the Diverse-broad responses from the training data. When the Deviant condition was left out, the resulting predictions of the responses in the left-out Deviant condition were consistently smaller than the actual responses (Fig. 13D, compare thin and thick red lines). On the other hand, when the Diverse-broad responses were left out, the predictions of the responses in the left-out Diverse-broad condition were consistently too high (Fig. 13E, compare thin and thick yellow lines).

## Discussion

### Summary of the results and relationships with previous studies

In this paper we studied the responses to low probability sounds in the auditory cortex of halothane-anaesthetized rats and the influence of the auditory context on these responses. The strength of the response to a given frequency depended on its probability of appearance in the sequence, as well as on the frequency composition of the rest of the sequence and on the ISI. Thus, when a frequency appeared in 5% of the tone presentations the responses were stronger than when it appeared in 95% of the tone presentations in 2-tone sequences (Figs. 1–7). This effect was large and robust for frequency differences of 21% and more, and significant at 10% (Figs. 6,7). Furthermore, responses depended on tone probability even with ISIs as long as 1800 ms, although this effect decreased somewhat at the longest ISIs (Fig. 8).

As a result, as long as the frequency separation between nearby tones was larger than about 20%, tones in low probability conditions elicited large responses (Deviant-alone, Deviant and Diverse-broad conditions). In contrast, high probability conditions (Standard and Equal-probability) gave rise to smaller responses. Even in a low probability condition, significant reduction in the responses could occur when the frequencies in the sequence were densely spaced (Diverse-narrow condition). This pattern of results was apparent in both LFP and MUA.

These results are consistent with previous studies of oddball responses. Ulanovsky et al. [5], [6] studied the responses of single neurons in cat auditory cortex to oddball sequences. The results of these papers are similar to those presented here, although the tone duration was 230 ms, substantially longer than the 30 ms used here. The ISI in the main data of the papers of Ulanovsky and colleagues, 730 ms, lies within the range of ISIs in which we showed significant effects, and the longest ISI at which they found significant effects was 2 s (while here we show significant responses at 1.8 s, but didn't test longer ISIs).

Lazar and Metherate [20] demonstrated SSA in epidural potentials recorded from rat auditory cortex with Δf of 100% and higher, but had full cross-frequency adaptation (‘spectral interactions’) with Δf of 11% or smaller. They used a slower rate of presentation, longer stimuli and higher probability of the deviant stimulus relative to those used in the current study. The pattern of their results is roughly consistent with ours, and the lower sensitivity in their study may be due to the large-scale averaging inherent in epidural recordings.

Malmierca and co-workers demonstrated significant SSA in rat inferior colliculus (IC) [7], [21] and in rat medial geniculate body (MGB) [10]. SSA with similar characteristics was also found in the MGB of mice [9]. Although adapting neurons were found in all IC and MGB subdivisions, SSA was much weaker and essentially non-existent at the rates used here in the ventral division of the MGB in rats [10]. It was mostly found outside the ventral division of the MGB in mice as well [9]. Given that the ventral division of the MGB is the major ascending input to the auditory cortex, these results suggest that much of the SSA measured in auditory cortex is actually generated in auditory cortex, with a possible small contribution from the MGB. Indeed, Szymanski et al. [22] showed that SSA in rat auditory cortex increased away from layer IV, suggesting an important contribution of cortical processing to cortical SSA. All of these studies used only the Standard, Deviant and Equal conditions, which makes it impossible to dissect out the contribution of pure adaptation to the Deviant responses, as we could do using the additional control conditions in the current paper.

Two recent reports demonstrated SSA in auditory cortex of awake rats as well. Von Behrens et al. [11] found SSA that was somewhat weaker than that reported here, but they used longer tones and longer ISIs than those used for most of the data reported here. Farley et al. [12] used, in addition to the Deviant condition, the so called ‘control’ condition of the MMN literature, which is equivalent to the ‘Diverse-broad’ condition used in this paper [17]. They found significant SSA to frequency shifts, and found the responses to tones in the Deviant and in the Diverse-broad conditions to be essentially equivalent on average. These findings are rather similar to those we report here.

Farley et al. [12] concluded that there is no deviance sensitivity in A1 of rats. However, the additional conditions we used in this paper allowed us to show that under a model of pure adaptation the responses in the Deviant condition should be smaller than in the Diverse-broad conditions (Fig. 9). Thus, the observed near equality of the responses in these two conditions (Figs. 5C and 5D) is actually surprising, and indicates the presence of true deviance sensitivity. We turn now to justify these statements.

### Mechanisms of SSA

Adaptation of the responses to tones of a given frequency is obviously induced by the presentation of the same tone frequency, but also by the presentation of tones of adjacent frequencies. Cross-frequency adaptation became significant at frequency separations between 21% and 44% (Fig. 7). This range is substantially narrower than the tuning width of either LFP or multiunit clusters (e.g. [23]), but is consistent with the width of the adaptation channels estimated by the models (Fig. 11B). Thus, adaptation occurs within channels extending about 1/3 octaves on either side of the center frequency.

Adaptation in narrow frequency channels, however, makes two predictions that are not fully born out even in with a cursory look through the data. First, the responses in all conditions should be smaller than the responses in the Deviant-alone condition; and second, that the responses in the Deviant condition should be smaller than in the Diverse-broad condition (Figs. 9A and 9B). The failure of both predictions is illustrated in Figs. 5C and 5D. First, a substantial number of recording sites had responses in the Deviant and Diverse-broad conditions that were larger than in the Deviant-alone condition. While these deviations could be argued away as noise in the measurement of the responses, the same figures also illustrate the near-equality of the responses in the Deviant and in the Diverse-broad condition. This near-equality leads to divergent estimates of the adaptation channel when using each of the two types of responses separately (Fig. 9C).

The leave-one-out procedure highlighted some additional features of the data. Four conditions resulted in consistent differences between the predictions to the left-out conditions and the actual measured responses in the same conditions. Two of these are easy to explain: leaving out the standard responses led to over-prediction of these responses, while leaving out the Deviant-alone conditions led to under-prediction of these responses. These effects presumably occurred due to the need to extrapolate the extreme responses (smallest response in the case of the Standard, largest in the case of the Deviant alone) based on the other responses, leading to ‘regression to the mean’ of the predicted responses. The reduction in the predicted responses to the Deviant-alone condition accounts for the effects of removing the Deviant-alone conditions from the training data on the estimated bandwidth (Fig. 12A). It is easy to see that these responses are an increasing function of the scale A and a decreasing function of the bandwidth σ. Thus, under-prediction of A (which is roughly the response in the Deviant-alone condition) requires a decrease in the bandwidth σ in order to account for the responses that remained in the training set.

More interestingly, leaving out the Deviant responses resulted in their under-estimation (Fig. 13D), but also to a significant increase in the estimated bandwidth of the models (Fig. 12A) and the largest reduction in the error of the fit among all left-out conditions for the LFP data (Fig. 12B). For the MUA data, the median reduction in the error was larger in the Diverse-broad condition, although non-significantly so; on the other hand, the 75% percentile of the error reductions of the Diverse-broad left-out models was much smaller than that of the Deviant left-out models. Removing the Diverse-broad responses resulted in the opposite trend: responses were over-predicted and the estimated bandwidth of the models became narrower.

Thus, it seems that the Deviant responses are somehow inconsistent with the rest of the data – removing them modified significantly the main parameter of the model, the bandwidth of cross-frequency adaptation, and reduced substantially the error of the fits. At the root of this inconsistency lies the fact that the responses to the deviant and diverse-broad conditions had about the same strength, but that both were typically somewhat smaller than the responses in the Deviant-alone condition. These findings are hard to accommodate in a model based on adaptation in narrow frequency channels (Fig. 9).

The contradiction between these two requirements is reflected in the predictions of the left-out conditions. In a model that was fitted without the Diverse-broad responses, there was a tendency of the estimated width of cross-frequency adaptation to be narrower, pulled in this direction by the inclusion of the Deviant responses in the training data. The reduction in the width of the adaptation channel resulted in predictions of the Diverse-broad responses that were too large, since most stimulus presentations in this condition fell outside the effective width of cross-frequency adaptation. The concomitant decrease in adaptation bandwidth and over-prediction of the Diverse-broad responses can be observed in Fig. 12A and Fig. 13E, respectively.

The opposite tendencies can be observed in the models in which the Deviant responses were excluded from the training data. The adaptation bandwidth was pulled to larger values by the reduction in the Diverse-broad responses relative to the Deviant-alone responses. As explained above, a wider adaptation bandwidth results in under-predictions of the deviant responses. The two effects can be observed in Figs. 12A and 13D.

### SSA, deviance detection and Mismatch Negativity

The inconsistency between the responses in the Deviant and in the Diverse-broad conditions suggests that somewhat different mechanisms operate in these two conditions. The Diverse-broad condition is (almost) symmetric with respect to all tone frequencies that appear in it; this is true also for the Diverse-narrow and Equal conditions. Thus, it makes sense to hypothesize that it is the composition of the oddball sequence, with its large asymmetry between the probabilities of the two tones, which engages special mechanisms. This assumption is further supported by Fig. 12B: the error of the fit was reduced more when removing the Deviant responses from the training data than when removing the responses to other conditions (at least for the LFP data, and for the more extreme cases of the MUA data), hinting at the fact that the Deviant responses are special. The contribution of the hypothetical mechanisms that are engaged in the Deviant condition to the response is large: adaptation in narrow frequency channels, fitted to all other conditions, accounted for only half of the average increase in Deviant relative to the Standard responses (Fig. 12D, compare thin and thick red lines).

Although we do not know what are the additional mechanisms that are engaged in the Deviant condition, the model presented here suggests one candidate: a dynamic, rather than fixed, adaptation bandwidth. The hypothesis is that the repetition of the standard stimulus causes a reduction not only in the responses to the standard, but also in its effects on the responses to other stimuli, causing the adaptation channel centered on the deviant frequency to become narrower. As a result, the deviant stimulus elicits similar responses to those of a stimulus with the same probability in the Diverse-broad condition, where such narrowing of the adaptation channel does not occur, but where the adaptation channel is stimulated much less. The enhanced responses in the Deviant condition may be interpreted as signaling the detection of the change, or deviance, that a deviant tone represents relative to the regularity set by the standard.

An important component of the auditory ERPs, the MMN, is believed to signal deviance detection in humans. There are many similarities between SSA in auditory cortex and MMN [13], suggesting that SSA lies upstream of MMN generation, although the early timing of SSA in auditory cortex indicates that the responses studied here are not those that directly cause the currents which are measured as MMN on the scalp. Furthermore, Farley et al. [12] failed to produce SSA in response to level deviants (but see [6]) and duration deviants, and showed that SSA in auditory cortex does not depend on NMDA receptors, while MMN does. These results emphasize the distance that separates SSA in auditory cortex and MMN.

In contrast with the negative results of Farley and co-workers, we demonstrate here that SSA in auditory cortex has one of the truly distinguishing characteristics of MMN [24], [25] – true deviance detection. Adaptation in narrow frequency channels should not be considered as true deviance detection, since it is sensitive to the rarity of the deviant but not to the regularity of the standard. The current results are the first demonstration that oddball sequences might engage true deviance-detection mechanisms, rather than only adaptation in narrow frequency channels, already at the level of auditory cortex. Therefore, these findings strengthen the case for SSA in auditory cortex as an important contributor to the generation of MMN.

## Materials and Methods

### Preparation

We used 26 adult female Sabra rats weighing 140–250 gm and 2 male Sabra rat juveniles, p27 and p34, for this study (Harlan Laboratories Ltd., Jerusalem, Israel). The joint ethics committee (IACUC) of the Hebrew University and Hadassah Medical Center approved the study protocol for animal welfare (protocols NS-06-10041-3, NS-08-11349-3). The Hebrew University is an AAALAC International accredited institute.

Animals were initially anesthetized with an intramuscular injection of ketamine (20–70 mg/kg, Ketaset, Fort Dodge Animal Health, Fort Dodge, IA) and medetomidine (0.05–0.5 mg/kg, Domitor, Orion Pharma, Espoo, Finland). Additional smaller doses of ketamine were administered as needed to maintain anesthesia during surgery. Surgical level of anesthesia was verified by pedal-withdrawal reflex.

The trachea was cannulated and the animal was fixed to a custom-made head holder [26], that left the scalp and ears free. The animal was ventilated through the tracheal cannula (10-15 mmH_2_O peak inlet pressure, 47/min, 15–30cc per stroke, 0.7–1.4 L/min) by a mixture of O_2_ and halothane (Rhodia Organique Fine Ltd., Bristol, UK) using a small-animal ventilator (model AWS, Hallowell EMC, MA), and a halothane vaporizer (VIP 3000, Matrx, NY). Once the animal was ventilated, ketamine anesthesia was discontinued, and halothane volume concentration was regulated around 0.5% to have a sufficient anesthesia depth. Throughout the experiment, respiration quality was monitored by continuously measuring the CO_2_ concentration in the tracheal cannula (Microcap, Oridion Medical Ltd., Jerusalem, Israel). The depth of anesthesia was judged by the lack of motion and resistance to the respirator, and levels of anesthetics and ventilation pressure were adjusted accordingly. Body temperature was monitored and maintained at 36–38°C using a rectal thermistor probe and a feedback-controlled heating pad (FHC Inc., ME).

The left temporal portion of the skull was cleaned from skin, muscles, and connective tissue. A craniotomy was performed over the estimated location of left auditory cortex – 2.5mm–6.5mm posterior to and 2mm–6mm ventral to bregma [27]. A copper wire hook implanted in the neck muscles was used as the electrical reference.

### Electrophysiological recordings

We recorded extracellularly from the auditory cortex using 1–4 glass-coated tungsten electrodes (Alpha-Omega Ltd., Nazareth-Illit, Israel), or a single micropipette. Metal electrodes were assembled together with separations of ∼600 microns. The electrodes were lowered into the cortex using a microdrive (MP-225, Sutter Instrument Company, Novato, CA).

The electrical signals were pre-amplified (×10), filtered between 3 Hz and 8 kHz to obtain both LFP and action potentials, and then amplified again, for a total gain of ×5000 (MCP, Alpha-Omega, Nazareth Illit, Israel), to yield the raw signals. The raw signals were sampled at 25 kHz and stored for off-line analysis. The analog signals were also sampled at 977 Hz after anti-aliasing filtering (RP2.1, TDT, Tucker-Davis Technologies, Alachua, FL), stored for LFP analysis, and used for online display.

### Sound stimulation system

All experiments were conducted in a sound-proof chamber (IAC, Winchester, UK). Sounds were synthesized online using Matlab (The Mathworks, Inc., Natick, MA), transduced to voltage signals by a sound card (HDSP9632, RME, Germany), attenuated (PA5, TDT), and played through a sealed speaker (EC1, TDT) into the right ear canal of the rat. In several animals, an acoustic calibration was performed as follows. A post-auricular incision was made and the meatus was cut as close to the skull as possible. A custom-made brass cone with speaker and microphone inlets was fastened so as to cover and seal the meatus in front of the tympanic membrane. The microphone (model EK-3133-000, Knowles, England) was previously calibrated against a calibrated condenser microphone (Type 2633, Brüel & Kjær, Denmark). Frequencies in the range of 1–64 kHz, with 20 frequencies per octave, were presented through the sealed speaker. When harmonic distortion in the microphone signal was larger than 0.5%, presentation level was reduced; the largest attenuation level at which harmonic distortion was lower than 0.5% was measured as well, and was found to be higher than 80 dB SPL for all tested frequencies. The intensity of stimuli during the experimental paradigm did not exceed this value. The calibration of the system was found to be stable across animals. The intensity deviations between the pairs of frequencies that are reported here did not exceed ±10dB near the tympanic membrane. In those experiments where calibration was performed, the speaker, the microphone and the brass cone were left in their position throughout the experiment.

For pure tones, attenuation level of 0 dB corresponded to about 100 dB SPL. Noise stimuli were synthesized at a spectrum level of −50 dB/sqrt(Hz) relative to pure tones at the same attenuation level.

### Experimental procedure

Recording sites were selected by their response to a broad-band noise (BBN). We searched for sites while continuously presenting 200 ms BBN bursts (0–50 kHz) with inter-stimulus time interval (ISI, onset to onset) of 500 ms and a level of 30 dB attenuation. The LFP responses were averaged online, and the electrodes were positioned at the location and depth that showed the largest evoked LFP responses over all the electrodes. Once selected, we validated and recorded the BBN responses of the recording site using a sequence of 280 BBN bursts with duration of 200 ms, 10 ms linear onset and offset ramps, ISI of 500 ms, and seven different attenuation levels, between 0 and 60 dB with 10 dB steps, that were presented pseudo-randomly so that each level was presented 40 times. The main data were collected if noise threshold level was at least 30 dB attenuation and noise-evoked potentials changed regularly with level; otherwise, the electrodes were moved to a different location.

Quasi-random frequency-level sequence of 777 tone bursts (50 ms duration, 5 ms onset/offset linear ramps, 500 ms ISI) at 37 frequencies (1–64 kHz, 6 tones/octave) and 7 attenuation levels (80–20 dB, 10dB steps, roughly corresponding to 20–80 dB SPL) were used to measure the frequency response area (FRA) of the recording site (3 presentations at each frequency-level combination). When the FRA was narrow or not smoothly graded with level, 370 tone bursts (300 ms ISI) at 37 frequencies (1–64 kHz, or narrower ranges when the FRA was narrow) were presented at a fixed attenuation level, in order to better characterize the frequency response in the level at which the main paradigm would be presented.

Once the recording site was characterized in terms of the best frequency and the minimum threshold, two frequencies, f1 and f2, were selected for the main experimental paradigm. Both tone had to be within the FRA, usually close and symmetric around the best frequency, and to have about the same response amplitude. The possible values of the difference between the two frequencies, defined as: Δf = f2/f1−1, were 10%, 21%, 44% or 96%.

We tested the responses to these frequencies in sets of up to seven different sequences. In most cases, each sequence consisted of 30 ms tone beeps with ISI (onset to onset) of 300 ms. A limited amount of data were recorded using sequences with ISIs of 500, 700, 1000, 1200 and 1800 ms. Each set of sequences was presented at a constant sound level, 20 – 40 dB above the minimum threshold of the recording site. The sequences are described in details in the Results section and in Figs. 1A and 3A. Each set of the seven sequences tested the responses to f1 and f2 in six different conditions.

In order to get comparable data from many paradigms in the same recording site, the sequences had to be as short as possible. Preliminary experiments showed that 25 presentations were enough to estimate an average response with a reasonable signal to noise ratio. Therefore, with the lowest presentation probability of each frequency being 5%, we used sequences of 500 tone beeps.

### Data Analysis

The data were analyzed with Matlab (The Mathworks, Inc., Natick, MA). To analyze LFP, all the responses to each frequency in a sequence were aligned on stimulus onset, and each was baseline-corrected by subtracting its average during the 5 ms interval starting at stimulus onset (response onset latency was always longer than 8 ms, and we wanted to adjust the baseline as close to the response onset as possible in order to avoid influence of slow waves in the signal). Response strength was quantified by the depth of the maximal (most negative) trough of the average response in the interval 0–70 ms after stimulus onset. We used a long time interval relative to stimulus duration (70 ms compared with 30 ms) because there was a tendency for adapted responses to be also delayed in time. The variability of the response was quantified by the standard error of the mean (s.e.m.) of the baseline-corrected responses at the time of the maximal trough of the average. Only sets with clear responses and a signal to noise ratio (response size divided by standard error) larger than 2 were included in the analysis.

To detect MUA, the raw signals were filtered between 200 and 8000 Hz, and large, fast events were marked as spikes. The threshold for spike detection was set to 12 times the median of the absolute deviations from the median (MAD) of the filtered voltage traces (corresponding to more than 7 standard deviations for Gaussian signals). This conservative criterion ensured that the detected events were indeed spikes and not random fluctuations of the baseline. The resulting spike trains were aligned on stimulus onset, smoothed with a 10 ms Hamming window, and averaged. MUA response amplitude was quantified by the peak response in the interval 0–70 ms after stimulus onset, without baseline corrections.

Responses of MUA were included in the data analyzed here only if they were clearly driven by the random-frequency tone sequences, and had statistically significant responses to the deviant-alone conditions in the 50 ms response interval starting at stimulus onset, compared with the 50 ms interval of spontaneous activity just preceding stimulus onset (2-tailed t-test, p<0.01). A few cases with very intense but short onset bursts resulted in no net increase of the average response rate during the 50 ms interval, but were nevertheless included in the data. On the other hand, this criterion rejected about 20% of the MUA data, in which responses were present but were late (deviant-alone response latency >50 ms). These late responses were not included in the population analysis.

## Supporting Information

Text S1 The relationships between the model used in the paper and models of synaptic depletion.
(DOC)
