# Supplementary material for: Stimulus-Specific Adaptation and Deviance Detection in the Rat Auditory Cortex
Source: PLoS One. 2011 Aug 10;6(8):e23369. doi: 10.1371/journal.pone.0023369 (PMC3154435; doi:10.1371/journal.pone.0023369)
Supplement: Text S1 — The relationships between the model used in the paper and models of synaptic depletion. (DOC) [file pone.0023369.s001.doc]

# Supporting information

# The relationships between the model used in the paper and models of synaptic depletion

We derive here the response of a neuron with depressing inputs to a sequence of tones. The model makes the following assumptions:

1. The neuron is described by one variable, *x*(*t*), which stands for the amount of synaptic resources available for generating a response at frequency $f_{c}$.
2. A stimulus with frequency *f* causes the release of a fraction of the synaptic resources, , where *U*(.) is a monotonically decreasing function of its argument.
3. Between stimuli, the synaptic resources recover exponentially to their maximal value (normalized to 1) with a time constant τ.

As a consequence of these assumptions, the response to a tone at frequency $f_{c}$ will be proportional to the amount of synaptic resources at stimulation time. We need therefore to derive the behavior of x(t) as a function of the stimulation sequence. We make a further assumption, which very nearly holds in the experiments conducted here:

1. The sequence of tones is i.i.d. multinomial, governed by a set of probabilities $p_{f}$, with a fixed interstimulus interval *d*.

Assume that frequency *f* is presented at time *t*. If the amount of synaptic resources just before stimulation is $x(t)$, the amount of resources after the stimulus is over is

$$x(t+)=x(t)-U(f_{c},f)x(t)=(1-U(f_{c},f))x(t).$$

Next, the synaptic resources undergo recovery until the next stimulus arrives, so that at the time of the next stimulation, $t+d$, the amount of synaptic resources is

$x(t+d)=1-(1-x(t+))e^{-d/\tau}$.

We denote $\delta=e^{-d/\tau}$. Therefore,

$x(t+d)=1-[1-(1-U(f_{c},f))x(t)]\delta$.

We iterate now: starting with $x(0)=1$, the tones occur at times $0,d,2d,...$ with frequencies $f_{0},f_{1},f_{2},$ and so on. We claim that the amount of synaptic resources at time *Nd* (just before the tone is presented) is

$x(Nd)=1-\sum_{n=1}^{N} \delta^{n}U(f_{c},f_{N-n})\prod_{k=N-n+1}^{N-1} (1-U(f_{c},f_{k}))$Eq. 1 .

Indeed, at $N=0$ the expression reduces to 1, and if it holds at time *Nd* then

$x((N+1)d)=1-[1-(1-U(f_{c},f_{N}))x(Nd)]\delta$.

Denote $x(Nd)=1-y$, where
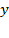
 is the second term at the right hand side of Eq. 1. Then

$x((N+1)d)=1-[1-(1-U(f_{c},f_{N}))(1-y)]\delta=1-\delta U(f_{c},f_{N})-\delta(1-U(f_{c},f_{N}))y$.

Reintroducing the definition of *y* we get

$x((N+1)d)=1-\sum_{n=1}^{N+1} \delta^{n}U(f_{c},f_{N+1-n})\prod_{k=N-n+2}^{N} (1-U(f_{c},f_{k}))$,

which is Eq. 1 with *N* replaced by $N+1$. By induction, Eq. 1 holds for all *N*.

Next, we calculate the expected value of $x(Nd)$, which is proportional to the expected size of the response to a tone of frequency $f_{c}$ at time *Nd*. Here we consider $U(f_{c},-)$ as a random variable. By assumption 4., $U(f_{c},f_{k})$ is independent of $U(f_{c},f_{n})$ for $n\neq k$. Therefore, the expectations of the products factor into the product of the expectations of each individual term. Denote $U=\left\langle U(f_{c},f)\right\rangle_{f}=\sum_{f} p_{f}U(f_{c},f)$; from Eq. 1 we get

$\left\langle x(Nd)\right\rangle=1-\sum_{n=1}^{N} \delta^{n}U(1-U)^{n-1}$.

This expression converges, for large *N*, to our main result,

Eq. 2 $\left\langle x(Nd)\right\rangle=1-\delta U\frac{1}{1-(1-U)\delta}=\frac{1-\delta}{1-(1-U)\delta}$.

Eq. 2 is the model prediction for the response given *U*. What are its relationships with the model we used in the main paper? The average *U* is functionally identical to the average we use in the main paper, $\sum_{f} p_{f}K(f_{c},f)$. Thus, the kernel used in the model is identified here essentially with the fraction of synaptic resources available for producing responses at frequency $f_{c}$ which are used when presenting frequency *f*. On the other hand, the adaptation depends on this average differently than in the main paper, where we used $AB^{U}$ instead of Eq. 2. However, since we are interested here in a restricted range of values for *U*, these two functional forms are similar to each other. Both are decreasing functions of *U* and both are bounded in the range of *U* relevant for the fitting process $(0.05<U<1)$. For a given value of *δ*, it is possible to find parameters for the exponential model that approximate Eq. 2 quite closely. This is illustrated in the Fig. A1, in which we used $\delta=0.6065$, corresponding to $d/\tau=0.5$. The green thick line represents Eq. 2 as a function of *U* over the range [0.05 1], while the thin blue line represents an approximation using the exponential form, with $A=0.924$ and $B=0.395$. These values are quite typical: *A* was usually close to 1, while *B* is about the relative response level of the standard. The relative difference between the two curves is less than 5% except close to 1, where is increases to about 7%.


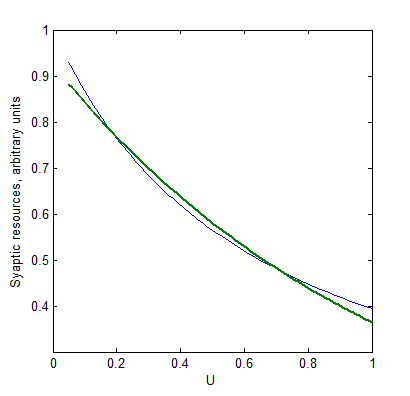


*Figure S1: comparing the synaptic depletion model with the exponential model used in the main text*

Numerically, Eq. 2 has the drawback of having a singularity which may come into play when fitting experimental data. It is easier to fit the exponential model since it doesn't have any inherent singularity. We therefore selected to fit the numerically more stable exponential form of the model in the main paper.
